# Social cognitive impairment and autism: what are we trying to explain?

**DOI:** 10.1098/rstb.2015.0082

**Published:** 2016-01-19

**Authors:** Susan Leekam

**Affiliations:** Wales Autism Research Centre, School of Psychology, Cardiff University, Park Place, Cardiff CF10 3AT, UK

**Keywords:** autism, theory of mind, social attention, domain specificity, horizontal and vertical integration

## Abstract

Early psychological theories of autism explained the clinical features of this condition in terms of perceptual and sensory processing impairments. The arrival of domain-specific social cognitive theories changed this focus, postulating a ‘primary’ and specific psychological impairment of social cognition. Across the years, evidence has been growing in support of social cognitive and social attention explanations in autism. However, there has also been evidence for general non-social cognitive impairments in representational understanding, attention allocation and sensory processing. Here, I review recent findings and consider the case for the specificity and primacy of the social cognitive impairment, proposing that we should focus more explicitly on clinically valid features for insights on the integration of ‘social’ and ‘non-social’ cognition.

## 1. Introduction

Despite being one of the most heritable of neurodevelopmental conditions, autism spectrum disorder continues to be defined as a behavioural syndrome that is based on clinical information from a child's developmental history and current behaviour. The diagnostic criteria are diverse, spanning not only the social domain (nonverbal communication, social reciprocity and peer relationships), but also behaviours in the non-social domain (restricted, repetitive, routinized behaviour and sensory reactions) [1]. However, there is surprisingly little research on the nature and potential reason for this co-occurrence between social and non-social symptoms. Instead, social cognitive research in autism in recent decades has focused on separating out the social impairment and its underlying cognitive or biological mechanisms. In this paper, I review recent findings that address the issue of specificity of the social cognitive impairment and propose that a domain-general explanation of these findings will help us to refocus attention on clinically valid features in both social and non-social domains.

Traditionally, theories of autism have been shaped by the pursuit of two traditional and interrelated goals. These are the goals of horizontal and vertical integration [2]. Horizontal integration refers to the way that a disparate set of behaviours or cognitive traits are to be understood relative to one another. The goal of horizontal integration is often pursued by making the assumption that certain cognitive/behavioural features are ‘primary’ or ‘core’. Other features are explained in relation to them in a secondary capacity, either as a downstream consequence or else as being incidentally associated and not specific to autism. Vertical integration refers to the way in which such traits are to be understood in terms of underlying neurobiological systems or processes and has been viewed as a particularly important goal for neurocognitive research [3,4]. The selection of primary features at the horizontal level has fuelled the quest for vertical integration, with scientists searching for abnormalities in brain regions or networks that underlie such core, primary features. Hence, assumptions governing the selection of clinical and cognitive features that are primary and specific to autism have led to the foregrounding of particular behavioural and cognitive symptoms over others, and directed the way that autism has been researched and understood.

## 2. A primary social cognitive impairment?

Within the history of autism research, there have been changing views about what should be considered to be the primary impairment. Kanner originally proposed in 1943 that an affective disturbance of children with autism was the primary impairment [5]. There followed a period in which sensory-perceptual and repetitive motor behaviour impairments were considered to be key factors [6] leading to the cognitive hypothesis by Hermelin and O'Connor in the 1970s that children with autism have difficulty in recruiting sensory input to make perceptual discriminations [7].

The arrival of domain-specific social cognitive theories changed this focus, postulating a ‘primary’ impairment of cognition specifically in the social domain described as a ‘theory of mind’ [8] or ‘mentalizing’ [9] impairment. This approach focused a growing new interest in cognitive explanations of autism more generally [10], towards a more specific account that targeted the core social and communication symptoms. This approach also had appeal for the goal of vertical integration in setting out an agenda that could causally connect diverse biological abnormalities to clinical behavioural symptoms by means of a simple cognitive mechanism designed for inferring mental states [9,11]. Currently, the theory of mind impairment still continues to be proposed as an important cognitive mechanism that can explain some key social communication functioning difficulties of autism [11–13].

The claims for a domain-specific cognitive impairment in autism began with evidence from Wimmer and Perner's false belief task [14] which was considered the ‘litmus test’ of theory of mind in that it aimed to test understanding about another person's mental representation of a situation independently of the current situation in the real world. In this task, children observe a scenario in which a protagonist puts an object into location A and leaves the room. The object is then unexpectedly moved to location B in his absence. Typical 4-year-old children correctly predict that on his return, the protagonist will look for the object where he left it, while younger children and also children with autism predict that the protagonist will look where the object is now. A landmark study by Baron-Cohen, Leslie and Frith in 1985 was the first of many studies to show that children with autism often fail or are extremely developmentally delayed in passing this task [15].

## 3. A domain-specific impairment in understanding mental representations?

Empirical evidence of false belief difficulty in children with autism has driven forward the quest to identify the nature and origins of this key social cognitive impairment. A crucial question is whether the impairment in attributing false beliefs is a domain-specific or domain-general capacity. This is different from the question of whether false belief impairment is specific to autism. Instead, the issue here is whether the cognitive requirements of this task belong specifically to a class of social cognitive or mentalizing processes rather than to general high-level cognitive or lower level perceptual processes. Those taking the domain-specific approach have argued that there is a dedicated, innate theory of mind system with specialized mechanisms that process mental representations about one's own and other people's beliefs [8,16]. On the domain-general side of the debate, the proposal has been that false belief understanding develops gradually through the development of general cognitive functions. For example, children develop understanding of alternative representations of the world [17,18] and improve in their executive control skills of cognitive inhibition and working memory, enabling the development of both mental state and non-mental state reasoning.

The debate between domain-specificity and domain-generality of false belief understanding was first tested empirically by comparing false belief tasks [14] with a false photograph task testing understanding of representation in the non-mental domain. In contrast to poor performance on the false belief task, children with autism excelled on the false photograph task [19]. However, the ‘formally identical’ nature of the false belief and false photograph tasks [20] was challenged on the grounds that a ‘false’ photograph is not actually ‘false’ but an outdated and true representation of an earlier situation [21]. In contrast, the false belief is a misrepresentation of the current reality. In order to properly test the domain specificity debate therefore, another task was devised—the false sign task [22], which like the false belief, misrepresents current reality. In the standard false sign task, a scenario is enacted in which children see a signpost that indicates an object in location A. For example, an arrow-shaped sign indicating an ice cream van points to its location at the village playground but then the ice cream van is moved to location B, the church, and children are asked, ‘Where does the signpost show that the ice cream van is?’

The results of a series of studies with typically developing children showed that performance on the false sign task strongly correlated with performance on the false belief task [23,24], even when age and performance on the false photograph task was controlled. Importantly, training studies with typically developing children aged 3–5 years also demonstrated that learning in one false representation task is potentially transferable to the other task [25]; by contrast, performance on the false photo and false belief tasks are not transferable [26]. The first study that was carried out with children with autism used a different, but structurally similar (false signal) task. Results showed that 10-year-old children with autism, matched for mental age with typically developing children, performed as poorly on this signal task as they did for the false belief task [27]. However, given that the false sign/signal tasks required complex language processing skills, it is possible that poor performance might be explained by this factor. The subsequent design of a new non-verbal false sign task, therefore, offered the opportunity to test false sign understanding without complex language demands or the need to inhibit where an object really is.

A non-verbal false sign task by Iao and co-workers [28] used a non-verbal version of the false belief task [29] that had previously shown selective impairments in children with autism compared with children who had Specific Language Impairment [30]. In this non-verbal false belief task, an object's location is hidden to the child and the child has to find the object. In a video version of this task [31,32], an object was placed in one of two boxes. A woman in the film saw where it was hidden, while participants did not. The object's location then continued to be unknown to the participant throughout the test trial. For the test trial, the woman then left the room and the boxes were swapped in her absence, creating a false belief in the woman. When she returned, she briefly placed a marker on the box to indicate the location that she thought contained the object, and removed the marker again. The participants then had to locate where the object really is, which required taking into account the box swap and the woman's resultant false belief to correctly locate the object in the box that the woman had not marked.

Performance on this task [31] was compared with an equivalent non-verbal false sign task. An electrically operated arrow-shaped signpost was positioned on a table between two boxes. In a brief training phase, children were familiarized with the operation of the signpost and quickly acquired the contingent link between the activation of the signpost direction and the location of an object in a box. In the test phase, as in the false belief task [32], an object was hidden in one of two boxes; the child did not know in which box the object was hidden. Participants could see the boxes on each side of the signpost but the signpost was covered by a screen. The signpost turned behind the screen towards one of the two boxes (the participant heard the usual sound of its activation but could not see or identify which box it turned to). The signpost was then switched off so its direction remained fixed. The locations of the two boxes were swapped. The screen was then briefly removed to reveal the signpost's direction and covered again. In order to correctly locate the object in the box, participants had to take into account the direction of the false sign and the swap of the boxes. Results showed that children with autism (verbal age 4–10 years) performed like the younger 3- to 5-year-old typically developing children. They failed both the false belief and false sign tasks while older 5- to 7-year-old children typically passed both. Additional experiments demonstrated a close correspondence between performance on non-verbal and standard verbal versions of both tasks [31]. Furthermore, performance on executive functioning control tasks showed that children with autism competently grasped other cognitive demands of the task including being able to inhibit a prepotent response and being able to use working memory to coordinate and recall the information components. Therefore, we argue that this evidence joins a growing body of other evidence (for example research measuring understanding of identity [33,34]) which supports the case for a domain-general difficulty. Hence understanding of belief does ‘not develop within an isolated domain but in unison with other domains that share needed conceptual abilities' [33].

## 4. A domain-specific impairment in implicit theory of mind?

How does the evidence for a domain-general impairment in understanding false belief, compare with recent evidence from several studies that measure *implicit* false belief reasoning? The two-system interpretation of theory of mind described by Apperly & Butterfill [35,36] provides a distinction between fast, automatic implicit processes and later developing, slower and more effortful explicit cognitive processes. Evidence of children's anticipatory looking behaviour during false belief tasks has been used to make the proposal that even typically developing infants can correctly ascribe implicit false belief even though they cannot give correct answers to direct questions in standard false belief tasks. By contrast, individuals with autism consistently have difficulty even on implicit false belief tasks.

In a series of implicit false belief studies, Senju and colleagues used an anticipatory looking paradigm [37], with individuals who had autism including adults [38], children aged 6–8 years (verbal age 3–12) [39] and 3-year-old siblings at risk of autism and other conditions [40]. In the Southgate–Senju task, participants watched a video in which a puppet placed the ball in one of two boxes, overseen by an actor. However, when a phone rang behind her, the actor turned behind and, while her back was turned, the puppet transferred the ball to a second box, then removed it, and then disappeared altogether. Participants were then presented with an audio-visual cue. They had previously learned in a familiarization trial that this cued a window to open and the actor to reach her hand through the window to retrieve the object. Results showed that even before the window opened, the typically developing 2-year-olds made correct anticipatory looks to the window above the box where the woman last saw it, even though in reality there was no object in either box; individuals with autism showed no bias for looking towards either box.

How should we compare the poor performance of individuals with autism on the Southgate–Senju task and the equally poor performance on the non-verbal false representation (false belief and false sign) tasks described above? Different explanations seem to be required. Although all these tasks were non-verbal and were designed to control for difficulties in language and executive functioning demands, if we follow the interpretation of the two-system account above, only the Southgate–Senju task qualifies as a candidate for implicit theory of mind (fast, automatic) while the other tasks would count as explicit (slow, effortful) tasks. In support of this conclusion, a series of anticipatory looking and explicit reasoning studies carried out by Low [41] with typically developing children showed that while implicit knowledge (anticipatory looking) was related to explicit reasoning, the capacity to pass explicit false-belief tasks was exclusively related to other skills such as higher order cognitive flexibility, representational understanding and complex language processing.

The results of ‘implicit’ theory of mind studies then may lead us to an assumption that individuals with autism have a problem with attributing belief spontaneously, in a rapidly changing social situation [12]. However, a major criticism of the Southgate–Senju task is that it does not actually provide evidence of the attribution of false belief in typical children [42]. Nor does the task necessitate the attribution of more simple mental states such as ‘know’ or even ‘see’ [42,43]. Instead it is possible to use anticipatory looking to ‘pass’ this task, by means of ‘behaviour rules’. These behaviour rules include keeping track of the direction in which agents look and forming an ‘experiential record’ of how agents behave [44]. One interpretation of the Southgate–Senju results therefore could be that individuals with autism had difficulty in tracking an agent's looking behaviour to different locations. By contrast, typically developing infants were able to quickly track the actor's head direction (indicated by the angle of the peak of the actor's visor). By tracking this association between left/right head direction and linking it to object location in the test trials, the typically developing child could consolidate a learned association already established in the four previous familiarization trials.

Nevertheless, even though this task does not provide evidence of attribution of ‘false belief’ or any other mental state for that matter, one might argue that the capacity to pass the task by tracking social cues highlights the potential power of social attention for typically developing children and the absence of this capacity for children with autism. Interestingly, however, the Southgate–Senju eye-tracking data do not fully support the hypothesis that children with autism fail to correctly anticipate due to failure to track where the actor is looking. Results showed that although participants with autism did show less spontaneous gaze checking of the actor's face, when this was statistically controlled for in the analysis, the group difference in anticipatory looking towards the correct box still remained [38,39]. Several other interpretations are possible for the pattern of performance. One possibility that I expand on below is that the individuals with autism had difficulty spontaneously allocating attention to the box when the connecting link between social cue and target object was disrupted. Another proposal made by Heyes [43] is of memory interference as children were distracted by the telephone ringing and head turning and failed to encode or recall the object's last location. The typically developing children resorted to looking to the location where they themselves had last seen the object and individuals with autism responded randomly with no reliable basis for predicting which window to look towards.

Heyes argues further that the bias to track human directional movement does not need to rely on a domain-specific social mechanism; non-social directional cues such as arrows also capture and direct attention to the same extent as heads and eyes [45]. When tasks are designed to test implicit theory of mind (in this case, the mental state of ‘seeing’) by including control tasks such as a directional arrow cue or a non-human agent, no differences between social and non-social conditions are found. Typically developing adults [46] and even adults with autism [47] perform similarly in both the conditions. To date, however, direct tests of the domain-specific hypothesis of implicit mental states understanding (e.g. belief, seeing and knowing) are few and have been confined to able adults. Meanwhile the domain-specific view has been generally accepted, particularly in the form of the impaired social attention hypothesis of autism. I review evidence for the social attention hypothesis below, before returning to domain-general approaches to autism.

## 5. An impairment in social attention?

So far, I have made the case that understanding of ‘explicit’ representational false belief involves an appreciation of the relationship between a representation and what it represents [17] and argued that false belief difficulty in children with autism reflects a domain-general impairment that applies to both mental and non-mental representations [31]. I have also supported the ‘lean’ interpretation [42,44] that correct anticipatory looking during ‘implicit’ false belief tasks does not provide evidence of ability to attribute beliefs or possibly any other mental state. Instead, the lack of correct anticipatory looking in these tasks might be explained by an inability to keep track of others' behaviour. This might be because of a lack of social attention or possibly because of a domain-general impairment that is not specific to social stimuli.

While domain-specific explanations propose innate, maturational, mechanisms that are specialized for handling mental representations [8,16,48], social attention accounts propose that reduced social attentional biases lead to reduced opportunities for experiential learning. These accounts have focused on ‘low-level’, bottom-up social cognitive processes, and their arrival has corresponded with a move towards more social constructivist and social cultural explanations of autism. Taken together, social attention accounts propose that children with autism fail to preferential orient to people due to impairment in the developing social brain network responsible for the processing of faces, human voices and biological motion, and impaired social reward preferences [49–51]. The resultant impoverishment in social information input, lack of social experience and reward from social engagement and lack of coordinated joint attention activities with others then has a cascading or downstream effect that compromises the development of cognitions such as theory of mind [52–54].

Although evidence for social orienting impairments in infants and in older children and adults has been extensively cited, it is important to remember that poor social gaze to faces, social initiation and lack of gaze following and pointing are already common clinical symptoms of autism by the time of diagnosis at 2–3 years, helping to assign children to the grouping of autism. If the goal is to provide an underlying explanation for these symptoms, it is important to avoid the circularity in simply re-describing them. One approach that helps to reduce this circularity is a longitudinal approach, which aims to identify social-specific behavioural or neural markers in the first year of life as independent predictors of diagnostic symptoms before these diagnostic symptoms actually appear. Another approach is to use experimental tasks that include both social and non-social stimuli.

Surprisingly, longitudinal findings taken from a range of measures of social orienting and social reward indicate very few differences between at-risk infants who are later diagnosed with autism, and typically developing infants in the first year of life. A recent review reported that basic aspects of orienting and reward (e.g. scanning of faces, preference to look at the eyes, response to still face) are intact in at-risk infants when they are six to nine months old [55] and even as young as two months [56], contradicting claims of an innate social orienting or theory of mind impairment from birth. Evidence from event-related potentials (ERP), at six to nine months old, do indicate failure to distinguish gaze direction [57], a result that is not replicated in terms of impaired behavioural gaze-following however until after 12 months when infants followed gaze but looked less to the gazed-at object. As the control tasks in these ERP studies did not include non-social directional cues it is not clear if the earlier difficulty reflected a general or specific response to a directional cue.

The limited range of evidence supporting both social and non-social impairments makes it difficult to draw strong conclusions. The findings above do not show that there is early social attention impairment in the first year of life in children who are later diagnosed with autism. This indicates that the domain-specific social attention account of innate social orienting or early learning in the first year is unlikely to be supported [58]. Other longitudinal evidence taken from typically developing children does show that anticipatory looking (‘implicit theory of mind’) at 18 months predicts false belief reasoning at 48 months [59]. However, even these findings may also need not necessarily indicate conceptual continuity. Awareness of ‘behaviour rules’ in infancy that someone will look where an object is last seen may be useful for building the reconceptualization at an older age that someone will think an object is where he or she last saw it and will look where he or she thinks it is. However, in tasks involving locations, it may be that it is possible to also draw information from domain-general direction cues as described above. This is indicated by the finding that significant longitudinal relationships between 18-month-old implicit anticipatory looking and 4-year-old explicit false belief were found only for the location tasks and not for the contents-identity false belief tasks [59].

Experiments with older children and adults have used structured Posner-type gaze-cueing paradigms, in which a central face cue looks left or right followed by a peripheral target stimulus appearing to the left or right. Social-specific impairments have not been found for the autism group where non-social arrow cues provide a control condition [60,61]. However, autism participants tend to respond more slowly in both conditions than ability matched control groups. These and other studies (e.g. [62]) reveal that the gaze-following capacity itself is not missing. Many individuals with autism follow gaze, especially when an instruction is given. By pre-school age, they can make fine discriminations based on the angle of the other person's eyes [63], yet tend not to spontaneously follow gaze unless given an explicit verbal or sensory cue [64] and have difficulty particularly at low developmental ages and when there is no visible target to ‘anchor’ their gaze [65]. Such reliance on predictable cue-target links might indicate difficulty in the spontaneous initiation and allocation of attention.

Evidence of atypicality in attention allocation can be seen in free-viewing tasks even with adults, for example in the vanishing-ball illusion. In this task, a magician throws a ball into the air twice. His social cues misdirect one's expectations and attention so that when he makes a third throw, this time a fake throw, we ‘see’ a ball vanish in the air. Eye-tracking findings in adults with autism viewing this illusion [66] showed that contrary to the predictions of the specific social attention hypothesis, they followed social cues and had typical patterns of fixation when looking at the magician's face and eyes. However, they were slower to launch their first eye movement to the face and had difficulty in fixating the fast-moving ball that he was looking at. Several other studies using either eye-tracking measures or electrodermal measures of non-social stimuli [67,68] have reported delay in responses to the first presented stimulus. These results are consistent with results from Senju's [38] adult findings for the ‘implicit’ false belief task of a group difference in first eye fixation, but the findings here suggest a more domain-general difficulty that is not specific to social stimuli.

Atypicality in sensory-perceptual and attentional processing is not only evident in adults, it is also evident in infants. Longitudinal findings of at-risk infant siblings in the first year of life who develop autism, show reduced visual fixation durations in spontaneous looking to a range of object stimuli at six to nine months old, indicating atypical attentional deployment at a very early age [69]. Other studies show enhanced visual search ability at nine months in the detection of target items from an array of distracters [70]. By contrast to this superior visual search, slowed disengagement of attention has also been found in 7- to 14-month-olds [53] although at least five studies have not reported atypical disengagement in older children [71]. A critical factor affecting disengagement performance is likely to be the stimulus presented [72]. Dynamic moving stimuli of abstract form and strong colour and that have high stimulus interest value to the participant will increase the slowing of disengagement [73].

Recent domain-general approaches focus on atypical perception, attention, sensory modulation and arousal [74,75] and aim to explain both diminished [76] and enhanced [75] abilities in individuals with autism at low and high levels of processing. For example, reduced multisensory facilitation [77] and difficulties with competing information processing demands may help explain not only performance on particular visuo-spatial tasks but also on high-level reasoning tasks (e.g. false belief tasks).

Sensory symptoms across all modalities are highly prevalent in autism [78] and delays in the allocation of attention may be exacerbated particularly when deviant or novel stimuli appear or in the presence of a strongly interfering stimulation [74]. Even after attention has been allocated to a particular stimulus, individuals with autism may have difficulty with modulating their attention, particularly when stimuli are complex and changing [79]. Understanding these kinds of difficulties is central to our understanding of clinical features of repetitive behaviour as well as to social-communication problems. Hermelin and O'Connor originally proposed that children with autism have difficulties in recruiting sensory input to make perceptual discriminations unless these are based on their own motor feedback [7]. New research aimed at measuring neural correlates of sensory [80,81] and also motor difficulties [82] may help to revive this account and develop an explanatory approach that connects sensory, motor and social/emotional clinical features.

## 6. Conclusion: what are we trying to explain?

Despite theoretical changes in the study of autism in the last few decades, the goals of horizontal and vertical integration continue to be pursued. Primary symptoms are still elevated above secondary symptoms and ‘core’ underlying biological mechanisms are still sought to explain these primary symptoms. These assumptions are part and parcel of the way that causal models have traditionally been formulated for developmental disorders, but in the new movement away from domain-specific, localized and innate explanations, I propose that we avoid the temptation to swing completely back in time to an exclusive focus on the sensory-repetitive clinical symptoms and non-social cognitive processing as primary symptoms.

Instead I suggest that we loosen the goal of horizontal integration beyond the grip of primary impairments, and consider other forms of integration that link across different types of social impairment and across social and non-social symptoms. Atypical behaviours in the social domain include difficulty in quite different sub-type areas of social functioning. These include non-verbal communication, conformity to social conventions, sharing and empathic relationships, and atypical social approach, e.g. aloof, passive or one-sided styles of interaction. How should these social sub-types be understood in relation to each other outside of a domain-specific explanatory framework? Furthermore, atypical behaviours in the social domain must, by necessity, co-occur with restricted and sensory features in order that any autism diagnosis can be given. How do different subtypes of social functioning interact with different subtypes of restricted and sensory functioning not only at behavioural but also cognitive and neural levels of description? Research has not yet scratched the surface of this question although some researchers are beginning to move in this direction by dissecting features within the social domain that cross several neurodevelopmental or psychiatric disorders [83]. Further work is needed across social and non-social domains if we are going to explain the social-cognitive impairment in autism in a domain-general context.

New ways of thinking about the goal of horizontal integration will lead to new ways of thinking about the goal of vertical integration. Neurocognitive models are now emerging that conceptualize alternative developmental pathways linking domain-general synaptic function to social and non-social autism symptoms [55] and incorporating heterogeneity at both neural and behavioural levels [75]. Developmental change is central to these models. Longitudinal evidence will be important in highlighting cases where there are early signs of sensory-perceptual and domain-general cognitive processing difficulties that do not develop into a clear-cut clinical presentation of autism. It has long been argued that developmental outcomes may not be determined by any particular critical underlying impairment [84] but instead that elements within causal relationships are co-actional, with factors having a dynamic impact on each other across time [85]. Therefore, we should retain a focus on the full range of clinically valid features of autism for insights on the integration of ‘social’ and ‘non-social’ cognition across development. Such insights are important not only for those who develop a clinical diagnosis of autism but also for those who do not.
